# Novel Approach to Periscapular Abscesses in a 2‐Year‐Old: A Case Report

**DOI:** 10.1155/cro/9645857

**Published:** 2026-09-02

**Authors:** Brendan Sweeney, Mackenzie Pargeon, Z. Deniz Olgun

**Affiliations:** ^1^ Department of Orthopaedic Surgery, UPMC Community Osteopathic, Harrisburg, Pennsylvania, USA; ^2^ Department of Orthopaedic Surgery, University of Pittsburgh, Pittsburgh, Pennsylvania, USA, pitt.edu

**Keywords:** abscess, infection, MRSA, pediatric, shoulder

## Abstract

**Purpose:**

We aim to present a novel approach to decompression of a periscapular abscess in the pediatric patient.

**Case:**

We present a 2‐year‐old male transferred to a Level 1 pediatric trauma center (PTC) from outside hospital (OSH) with MRSA positive blood cultures in sepsis. Before presentation to the OSH, the patient had several days of right upper arm pain with any movement and developed a fever the day before. Magnetic resonance imaging (MRI) of the right shoulder at PTC revealed subscapularis and supraspinatus abscesses. Utilizing a modified Judet approach, the supraspinatus abscess was directly decompressed. Through the same approach, a corticotomy of the scapular body was performed with a high‐speed burr, and the subscapularis abscess was indirectly decompressed. Two drains were placed, and appropriate antibiotic therapy continued.

**Results:**

The patient was discharged on postoperative day 3 after transition to oral antibiotics by infectious disease. The patient was followed up in pediatric clinic on postoperative day 15 without recurrence of infection and with full painless range of motion. At that time, the incision was well healed without drainage or erythema. The patient was continued on oral Bactrim for two more weeks. To date, the patient has had no recurrence.

**Conclusion:**

We present a viable option for decompression of periscapular abscesses in the young pediatric population when traditional approaches such as deltoid split or deltopectoral would not be possible given the altered anatomy of the young and immature patients.

## 1. Introduction

Pediatric patients present unique challenges when attempting to treat most pathologies given their differences in physiological responses and nonidentical anatomy when compared with fully mature adults. This is all the more applicable when discussing how to evaluate and treat infections in the pediatric population. Of all patients presenting for acute illnesses concerning for infection and requiring blood cultures, 1.2% are diagnosed with bacteremia. The primary cause for infection has historically been encapsulated organisms such as *Haemophilus influenzae* and *Streptococcus pneumoniae*. With improvement in *H*. *influenzae* vaccination, the primary organism has become solely *S*. *pneumoniae* with *Staphylococcus aureus* being the second most common [1, 2].

One of the key issues is determining which patients are presenting with potentially serious infections that may lead to bacteremia and sepsis. The current guidelines for blood culture collection in the pediatric population are stringent to decrease overuse and false positives that may lead to unnecessary use of antibiotics. This is especially true for patients in the intensive care unit with new fevers following prolonged treatment or recent surgical procedures. However, patients with infectious signs and symptoms, including fever, who present with other signs of sepsis should always have blood cultures drawn [3, 4].

Furthermore, patients with intra‐abdominal or respiratory infections tend to be more obvious to parents and outpatient providers than infections stemming from the musculoskeletal (MSK) system. Patients with MSK infections usually present within days of a direct injury to the extremity in question. In fact, classically, it has been described that 30% of patients with osteomyelitis have reported an acute trauma to the region within 1 week of presentation. Other common symptoms to look out for in a patient with extremity infection, including osteomyelitis, septic arthritis, and periarticular abscess, are refusal to bear weight on the extremity with associated fever [5].

In contrast to bacteremia from other bacterial species, *S. aureus* bacteremia presents with higher virulency and patients in higher severity of condition, with methicillin‐resistant *Staphylococcus aureus* (MRSA) causing the most concerning infection. The reason for increased concern with MRSA infection is that most MRSA infections require surgical source control, whereas methicillin‐sensitive *Staphylococcus aureus* (MSSA) infections can be treated with antibiotics if there is not an associated abscess formation. Likewise, osteomyelitis can also be treated with antibiotics alone if there are no associated subperiosteal abscesses [5, 6].

We present a 2‐year‐old male with several days of right upper extremity pain following a pulling injury on his arm by parents. He was initially seen at an outside hospital (OSH) emergency department (ED) with concern for nursemaid′s elbow. He was eventually found to have a fever and inability to bear weight on the extremity. Consequently, the patient became septic and bacteremic with MRSA due to a periscapular abscesses diagnosed with MRI of the right shoulder.

## 2. Case Summary

The patient initially presented to an OSH with right upper extremity pain and fevers of 1‐day duration. At the time, there was concern that the patient had an upper respiratory infection as well as an unrelated nursemaid′s elbow of the right upper extremity. The outside facility attempted closed reduction of the suspected radial head dislocation. He was then sent home from the ED that day and instructed to follow up with their primary care provider.

Two days after the initial evaluation at the hospital, the patient was brought back to the same ED with continued fevers and increasing right upper extremity pain and swelling. At this time, the patient met sepsis criteria, and blood cultures were taken. After 1 day in the hospital, the patient was found to be bacteremic with MRSA and required a higher level of pediatric care.

**Figure 1:**
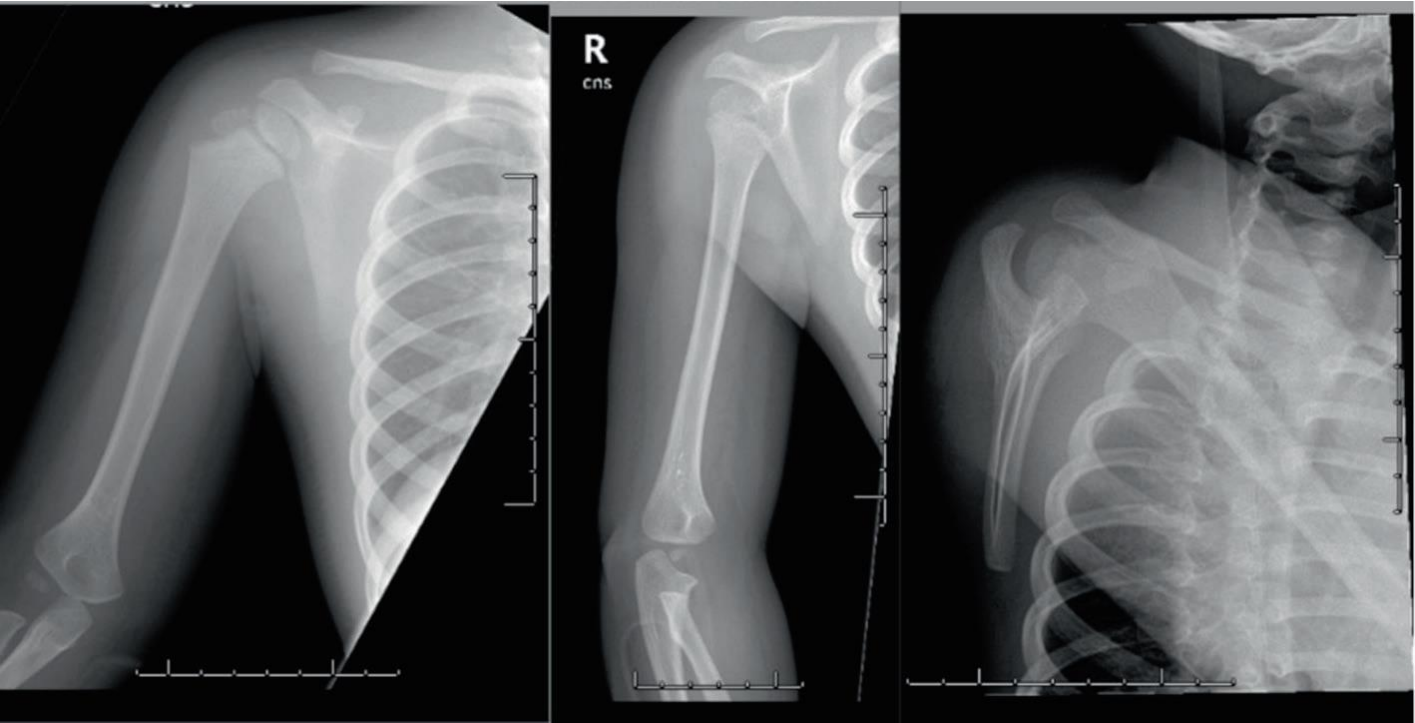
Radiographs of the humerus obtained on admission demonstrating no obvious signs of osseous involvement of the right shoulder.

The patient was transferred to our Level 1 pediatric referral center and admitted to the pediatric intensive care unit where he continued to receive broad spectrum antibiotics and supportive care. Here, he was found to have elevated inflammatory markers including a C‐reactive protein of 7.99, erythrocyte sedimentation rate of 29, and white blood cell count of 15.5. Orthopedic surgery was consulted for evaluation due to increasing swelling and redness of the right upper extremity. During initial evaluation by orthopedic surgery, the patient was noted to have painful, limited range of motion of the right shoulder. A radiograph was obtained of the humerus and can be seen in Figure 1, but demonstrated no obvious abnormalities. For this reason, an MRI of the right shoulder was obtained, looking for an infectious focus.

**Figure 2:**
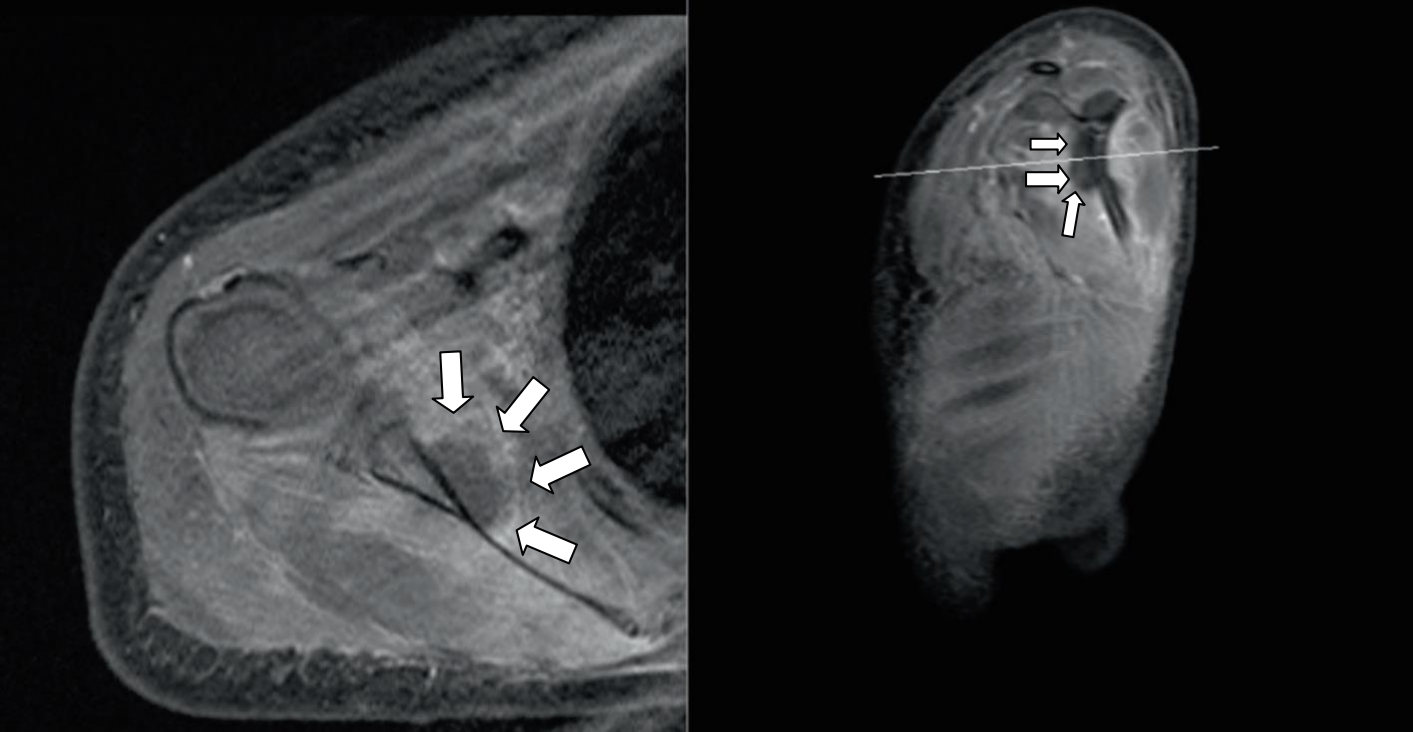
T1 fat saturation postcontrast MRI of the right shoulder with contrast demonstrating a midsubstance subscapularis abscess along the ventral border of the scapula measuring 2.6 by 1.6 by 0.8 cm.

**Figure 3:**
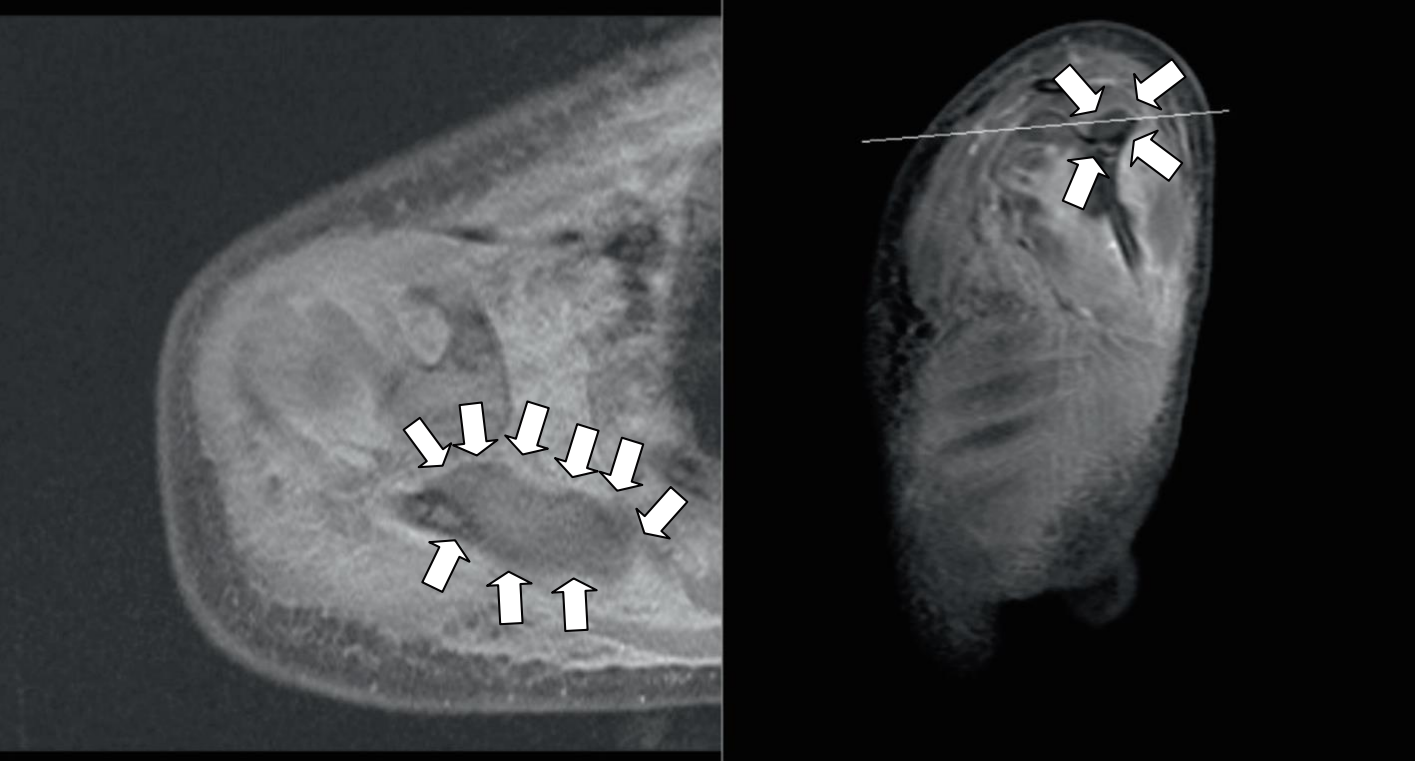
T1 fat saturation postcontrast MRI of the right shoulder with and without contrast demonstrating an abscess within the supraspinatus fossa measuring 3.6 by 1.5 by 0.9 cm causing significant myositis in the supraspinatus muscle belly. Abscesses outlined in white arrows.

MRI of the right shoulder revealed signal enhancement of the supraspinatus fossa as well as the subscapularis muscle belly concerning for two intramuscular abscesses which can be seen in Figures 2 and 3. The initial plan was to involve interventional radiology for percutaneous placement of drains.

However, the interventional radiology team ultimately decided the location of the abscesses precluded safe and adequate placement of drains. The orthopedic surgery team therefore proceeded with the procedure described below.

## 3. Operative Details

The appropriately anesthetized patient was positioned prone. A modified Judet approach to the scapula was performed utilizing a curvilinear incision along the spine of the scapula to the medial border and continued caudal to the inferior angle of the scapula. Sharp dissection was then carried through subcutaneous tissue down to muscular fascia where the posterior deltoid was identified and mobilized. The spine of the scapula was easily identified deep to fascia. A periosteal elevator was utilized to elevate the supraspinatus muscle from the supraspinatus fossa with immediate return of obviously purulent fluid. A sample of the fluid was obtained and sent for cultures. A thorough irrigation and debridement of the fossa was then performed utilizing Metzenbaum scissors to bluntly spread along the cavity, which resulted in additional expression of purulent discharge.

Attention was then turned to the infraspinatus musculature. A scalpel was used to identify the subperiosteal interval over the medial aspect of the scapula. The musculature was elevated off the infraspinatus fossa and reflected cephalad with care taken to prevent impingement upon the spinoglenoid notch and suprascapular nerve. A bony window of about was made using a high‐speed burr, which resulted in 5–6 cc of purulent discharge after breaching the anterior cortex. A Kerrison rongeur was then used to expand the cortical window to approximately 2 cm in diameter to allow adequate access to the subscapular musculature. The medial and lateral borders of the scapula were directly visualized. The corticotomy was then made approximately 2 cm medial from the lateral border and 1 cm inferior to the inferior border of the scapular spine to avoid the suprascapular nerve superolaterally while still allowing ample space from the medial border to avoid the spinal accessory nerve. With the corticotomy only 1 cm inferior to the scapular spine, the corticotomy was far superior to the inferior angle and prevented any disturbance to the growth of the scapula. Any additional bone bleeding was controlled with bone wax along the edges of the corticotomy.

**Figure 4:**
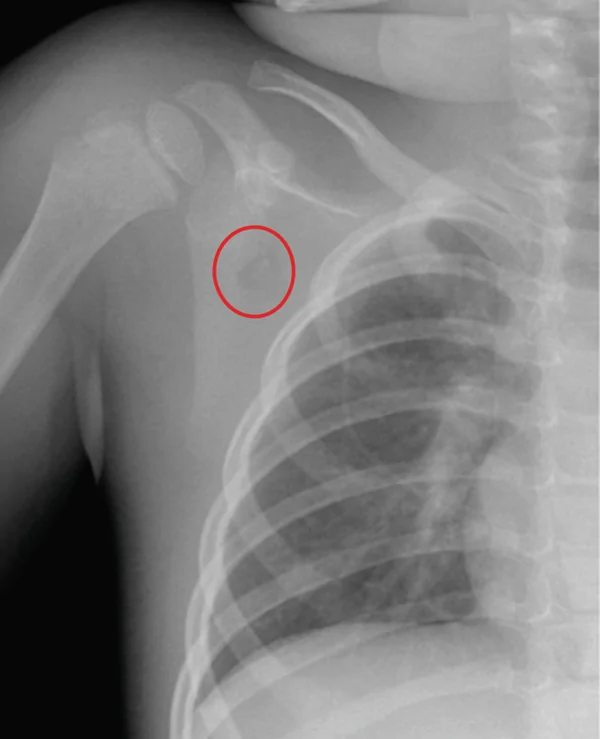
Section of anteroposterior radiograph of the chest demonstrating location of corticotomy (red circle).

**Figure 5:**
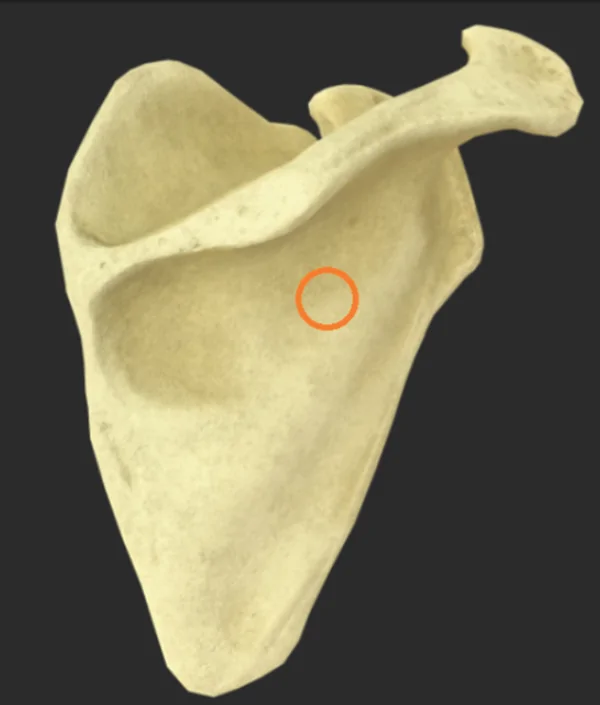
Diagram of adult scapula. Highlighted (orange) region is the approximate area of corticotomy.

A postoperative chest radiograph demonstrates the location of the corticotomy in Figure 4. An additional diagram of the scapula is seen in Figure 5 that shows a region of approximate location of the corticotomy highlighted in orange. An excisional debridement was then performed using rongeurs and curettes. Both bone and soft tissue specimens were obtained from this location and sent to cultures. The wound and abscess beds were then thoroughly irrigated and closed in sequential fashion.

The patient was returned to the PICU intubated and sedated and remained in this condition until postoperative day 1. The patient was continued on IV antibiotics with daptomycin to treat the MRSA‐positive intraoperative cultures. A detailed table of sensitivities and culture data is seen in Table 1. On postoperative day 2, the patient was extubated and downgraded to the medical floor. On postoperative day 3, the patient was found to be afebrile with decreased inflammatory markers (CRP of 1.93 and WBC of 9.1). He was deemed medically stable for discharge on postoperative day 3 after transition from IV daptomycin to PO Bactrim.

**Table 1:** Culture results and antibiotic susceptibility.

	*Staphylococcus aureus* isolated from anaerobic bottle (MRSA) (VITEK MIC—ug/mL [CHP]), method not specified	
**Ciprofloxacin**	**> = 8**	**Resistant**
Clindamycin	0.25	**Sensitive**
Daptomycin	0.25	**Sensitive**
Erythromycin	<=0.25	**Sensitive**
Levofloxacin	4	**Resistant**
Linezolid	2	**Sensitive**
Oxacillin	> = 4	**Resistant**
Rifampin		**Sensitive**
Sulfa/trimethoprim	<=10	**Sensitive**
Tetracycline	<=1	**Sensitive**
Vancomycin	1	**Sensitive**

## 4. Discussion

Managing pediatric patients requires careful consideration of various factors, particularly when addressing complex infections. In this case, a specialized approach was necessary to decompress the periscapular abscesses effectively. Given the complexity of the infection, a multidisciplinary team was engaged, including the pediatric hospitalist, pediatric intensive care team, infectious disease specialists, and the pediatric orthopedic team, to ensure comprehensive management. Interventional radiology was consulted to assess the feasibility of percutaneous drainage; however, due to the patient′s small size and anatomical constraints, the procedure was deemed too high‐risk to pursue.

As stated previously, the main concern when patients are bacteremic with MRSA is to obtain source control [1]. In complex patients with difficulty decompressing pyogenic myositis, there have been previously described reports that describe unique approaches in the past. Yilmaz described two cases of periscapular abscesses in pediatric patients, one 7‐ and one 5‐year‐old child, who underwent decompression of their abscesses using a direct posterior or deltopectoral approach, respectively [7].

In addition, to the care given to understanding infection, it is also imperative to identify the root cause of the infection. In young children, it can often be difficult to obtain a reliable exam in order to pinpoint the location of concern. For this reason, MRI is often employed. This comes with its own set of challenges, as young children often require sedation in order to keep the child still and decrease artifact; which was true for our case.

As of late, a new suggestion has been presented that may aid in the diagnosis of septic arthritis: ultrasound (US). Çolak et al. presented a case of septic arthritis in an adult shoulder that was evaluated with US and resolution of edema and effusion was followed using US. Additionally, Mısırlı et al. presented a case in which US was performed to identify and trend the resolution in symptoms from extensor carpi ulnaris (ECU) strain. These studies suggest that US may be beneficial in our understanding of complex infections of the MSK system, as well as present a cheap, point of care option for identifying joint effusion without the need for sedation as is the case with MRI in pediatric patients [8, 9].

Furuhata et al. described a case of complex abscess within the subscapularis muscle in a 67‐year‐old patient. In this report, they were able to decompress the abscess through a deltopectoral approach with anterior extension along the glenoid. They were able to fully decompress through the deltopectoral approach by elevating the subscapularis off the capsule using blunt instrumentation to puncture the abscess [10]. Additionally, McFarlane et al. performed a literature review on 17 cases of periscapular abscess that were all surgically decompressed in adult patients. A modified Judet approach was not utilized in any of these cases [11].

We described our approach as novel in the management of periscapular abscesses in a pediatric patient due to its efficacy in treating abscesses of multiple compartments of the shoulder girdle through a single approach. The use of corticotomy allowed for decreased dissection around the anterior scapula by keeping our dissection posterior. Although traditional surgical techniques, such as the deltopectoral approach described by Jagernauth et al. [12], have been effective in adults, they pose several limitations in pediatric patients due to differences in anatomy. In older or larger patients, a deltopectoral approach may have been a viable option, as seen in the first case of the Yilmaz case series [7]. However, in our case, abscesses located both dorsal and ventral to the scapula required a strategy that has not been well described in recent literature.

Although this manuscript is not the first to explore alternative methods for decompressing periscapular abscesses in pediatric patients, existing reports remain limited. For instance, Khaw and Faisham described a single‐incision technique from the posterolateral angle of the scapula for decompression of subscapularis and deltoid abscesses [13]. What further separates our approach from the others described was the use of a high speed burr and Kerrison rongeur to create a corticotomy within the body of the scapula. Given the patient′s age, care was taken to make the corticotomy medial and inferior enough to avoid the suprascapular nerve. Additionally, placing the corticotomy at the body of the scapula, well superior to the inferior angle and medial edge, limits the potential for growth arrest as the scapula primarily grows along these borders.

Our approach provided effective access to both infected compartments of the scapula from a single surgical position, optimizing the likelihood of achieving a curative outcome while minimizing the need for multiple anesthesia events. The patient′s rapid recovery and absence of recurrence further support the efficacy of this technique, underscoring its potential as a guideline for future procedural management of periscapular abscesses in very young patients.

The major limitations to our study were the lack of long‐term follow‐up and that this is a single case, case study. Our patient followed up at 2 weeks (postoperative day 15) and was doing well without major pain or signs of recurrent infection. To date, the patient has not followed up after a 2‐week postoperative appointment. Additionally, per chart review, the patient has not had any further ED visits or pediatric visits concerning recurrent MRSA infections. Additionally, the use of this approach in further cases to bolster our claims would help this study, but no other patients have presented in a similar fashion to attempt this approach again.

## 5. Conclusion

This report presents a novel surgical approach for the management of subscapularis and supraspinatus pyogenic myositis secondary to MRSA bacteremia in a 2‐year‐old pediatric patient. Our technique provided effective access to both infected compartments of the scapula from a single surgical position, facilitating thorough decompression. The patient′s rapid recovery and absence of recurrence support the efficacy of this approach in our patient and could potentially be utilized for future procedural management of periscapular abscesses in very young patients.

## Funding

No funding was received for this manuscript.

## Ethics Statement

IRB exemption was obtained from the host institution in accordance with the host institution′s protocols.

## Consent

Informed consent was obtained from the patient′s grandmother who is the legal guardian. The grandmother assented to use this case as a report at the time of the initial procedural consent and is included in the initial procedural consent form.

## Conflicts of Interest

The authors declare no conflicts of interest.

## Data Availability

The data that support the findings of this study are available on request from the corresponding author. The data are not publicly available due to privacy or ethical restrictions.
